# Relationship between household socio-economic status and under-five mortality in Rufiji DSS, Tanzania

**DOI:** 10.3402/gha.v6i0.19278

**Published:** 2013-01-24

**Authors:** Cornelius Nattey, Honorati Masanja, Kerstin Klipstein-Grobusch

**Affiliations:** 1 National Institute for Occupational Health, Johannesburg, South Africa; 2 Ifakara Health Institute, Ifakara, Tanzania; 3 School of Public Health, Faculty of Health Sciences, University of the Witwatersrand, Johannesburg, South Africa; 4 Julius Global Health, Julius Center for Health Sciences and Primary Care, University Medical Center Utrecht, Utrecht, The Netherlands

**Keywords:** inequality, socio-economic status, principal component analysis, mortality concentration index

## Abstract

**Background:**

Disparities in health outcomes between the poor and the better off are increasingly attracting attention from researchers and policy makers. However, policies aimed at reducing inequity need to be based on evidence of their nature, magnitude, and determinants.

**Objectives:**

The study aims to investigate the relationship between household socio-economic status (SES) and under-five mortality, and to measure health inequality by comparing poorest/least poor quintile mortality rate ratio and the use of a mortality concentration index. It also aims to describe the risk factors associated with under-five mortality at Rufiji Demographic Surveillance Site (RDSS), Tanzania.

**Methods:**

This analytical cross sectional study included 11,189 children under-five residing in 7,298 households in RDSS in 2005. Principal component analysis was used to construct household SES. Kaplan–Meier survival incidence estimates were used for mortality rates. Health inequality was measured by calculating and comparing mortality rates between the poorest and least poor wealth quintile. We also computed a mortality concentration index. Risk factors of child mortality were assessed using Poisson regression taking into account potential confounders.

**Results:**

Under-five mortality was 26.9 per 1,000 person-years [95% confidence interval (CI) (23.7–30.4)]. The poorest were 2.4 times more likely to die compared to the least poor. Our mortality concentration index [−0.16; 95% CI (−0.24, −0.08)] indicated considerable health inequality. Least poor households had a 52% reduced mortality risk [incidence rate ratio (IRR) = 0.48; 95% CI 0.30–0.80]. Furthermore, children with mothers who had attained secondary education had a 70% reduced risk of dying compared to mothers with no education [IRR = 0.30; 95% CI (0.22–0.88)].

**Conclusion:**

Household socio-economic inequality and maternal education were associated with under-five mortality in the RDSS. Targeted interventions to address these factors may contribute towards accelerating the reduction of child mortality in rural Tanzania.

Sub-Saharan African countries are confronted with a myriad of problems in their effort towards development. Prominent among them are diseases, poverty, and armed conflict. Diseases like malaria, diarrhoea, and the ongoing HIV epidemic are the major causes of under-five morbidity and mortality. Child mortality is a good indicator of child health and survival (1).

Globally, significant progress has been made in reducing mortality rates among children. Between 1990 and 2010, the under-five mortality rate declined by 35% – from an estimated 88 deaths per 1,000 live births to 57 (2). The global rate of decline has also accelerated in recent years – from 2.1% per annum during 1990–2010 to 2.6% during 2005–10 (2). The annual rate of decline in Africa – where almost half of all child deaths occur – increased from 1.8% during 1990–2010 to 2.8% during 2005–10. Despite this improvement, most countries in the region are unlikely to achieve the fourth Millennium Development Goal target of a two-thirds reduction in 1990 mortality levels by the year 2015 (2).

Bilateral donors such as the United Kingdom's Department for International Development have put improvement of the health of poor people as their top priority in the health sector (3), as has the World Health Organization (4) and the World Bank (5), which recognises low socio-economic status (SES) and avoidable child mortality as not simple inequalities, but inequities that are unjust and unfair.

Poor children are more exposed to risks of disease through inadequate water and sanitation, indoor air pollution, crowding, poor housing conditions, and high exposure to disease vectors (6). They are also more likely to have lower resistance to infectious diseases because they are undernourished (an underlying cause of about 50% of deaths in children younger than age 5) (7); to have diets deficient in one or more essential micronutrients (e.g. vitamin A, iron); to have a low birth weight as a result of poor maternal nutrition, infections during pregnancy, and short birth intervals; and to have recurrent disease episodes (6). The deprivation of poverty goes beyond low income. Low income is associated with lower levels of education, and lower education is associated with disease exposure. For example, in a poor household, knowledge can make the difference between taking advantage of piped water to wash hands and not doing so (8). Knowledge plays a role in, for example, securing a nutritious diet and making appropriate use of health care services (9). Poverty thus increases exposure and reduces resistance to disease, a synergy that contributes to the wide inequities in child survival.

A recent review suggests a linear relationship between maternal education and childhood mortality and the absence of a threshold effect: even a few years of maternal education make a difference (10). In the same review, poverty was suggested as the underlying factor in many cases of child death.

Although there have been improvements in the health status of Tanzanians, infant and child mortality is still high in rural Tanzania. In a poor rural area of Tanzania, the poorest children were 27% less likely to seek care from an appropriate provider than better off but still poor, and children from the poorest families were not as likely as their better-off peers to have received antimalarials for fever or antibiotics for pneumonia (11). The Tanzania second National Strategy for Growth and Reduction of Poverty 2010 (NSGRP-II) (12) document has a development vision to reduce under-five mortality from 154 in 2002 to 79 per 1,000 live births by 2010. Subsequently, in the most recent Tanzania Demographic and Health Survey Report (TDHS) (13), under-five mortality was reported to be 88 per 1,000 births, denoting a considerably decrease of under-five mortality in the period 2002–10. Still, the 2010 TDHS report showed marked differentials between wealth quintiles and child mortality, with under-five mortality declining gradually with increasing wealth in rural Tanzania.

Rufiji in the Pwani region is a relatively poor subsistence rural area in Tanzania. The Rufiji district is one of the six districts of the Pwani region, Tanzania. It has a population size of about 226,000 of which 87,000 (about 38% of the district) are under periodic surveillance (14). The Rufiji Demographic Surveillance Site (RDSS) commenced field operations in November 1998. The DSS approach involves periodic monitoring of households and members within households in cycles or intervals, known in the RDSS as ‘rounds’ every four months, that is, three times a year each. The RDSS collects information on demographic, socio-economic, and environmental characteristics of a population of about 87,000 people in 31 villages along the coastal area of Tanzania, south of Dar es Salaam in the Rufiji River basin. A team of trained fieldworkers went from household to household to collect this information in Swahili after obtaining informed consent from each household head. At first glance, the population of RDSS appears to be uniformly poor. However, within this community there are poverty gradients; hence, the focus in this current analysis is to investigate whether, among poor rural Tanzanian populations, wealth index as a proxy for SES and health inequality assessed by concentration index and poorest/least poor quintile mortality rate ratio is related to under-five mortality.

Although previous studies (15, 16) have suggested an association between SES and child mortality at RDSS, information on mortality differentials linked to health inequality could provide further evidence for targeted intervention towards reduction of under-five mortality.

## Methods

### Design and study setting

Based on the data from repeated four-monthly cross-sectional surveys, an analytical cross-sectional study was carried out including all households residing within the RDSS in 2005. Data were extracted from the RDSS database including information on all individuals, household head, household assets, and deaths that occurred in 2005.

The study sample comprised 11,189 children younger than age five on 31 December 2005 in 7,298 households in the RDSS for whom information on household SES characteristics were available. There were 289 under-five deaths in 2005; for the current analysis, information on 251 deaths was available.

### Assessment of SES

Socio-economic status was measured using an asset index, based on ownership of assets, water and sanitation facilities, power source, and housing quality as recommended by Filmer and Pritchett (1998) (17). Assets (Appendix A) were combined into a wealth index using weights derived through principal component analysis (PCA). The PCA involves breaking down assets (e.g. radio, bicycle) or household service access (e.g. water, electricity) into categorical or interval variables. The variables are then processed in order to obtain weights and principal components. Based on this index, SES of households was divided into quintiles (i.e. poorest, very poor, poor, less poor, and least poor) representing proxies for SES.

### Assessment of health inequality

Health inequality in under-five mortality was measured using the concentration index proposed by Wagstaff et al. (18). It is computed from the mortality concentration curve, which plots the cumulative proportions of children ranked by the household's SES against the cumulative proportions of under-five mortality. It estimates the extent of socio-economic inequality in mortality. The concentration index is similar to the relative index of inequality frequently used by epidemiologists (19). It takes values between −1 and 1. A value of 0 indicates equity in the health variable. A negative value indicates excess concentration of health among the poor, and a positive value indicates that the poor are less healthy than would be expected had the distribution been equitable. Then, we used the poorest/least poor quintiles mortality rate ratio (PPR) to compare rates prevailing in the poorest quintiles with those in the least poor quintiles. This approach ignores the information contained in the middle three quintiles.

### Other variables

Further explanatory variables included in the analysis were maternal education, maternal age, maternal occupation, maternal marital status, and sex of child. Information on birth weight and distance to facility and nutritional status, known risk factors of under-five mortality, were not collected at the RDSS and hence were not available for analysis, presenting a limitation of this study.

### Mortality rate

The under-five mortality rate was measured by dividing the total number of deaths in a wealth quintile by the calculated person-years observed (PYO) for all children younger than age 5 in that particular quintile for the year 2005. It was expressed per 1,000 PYO. Infant mortality rates were calculated the same way by wealth quintile.

### Statistical analysis

The PYO from 1 January 2005 to 31 December 2005 were computed for all children younger than age 5 residing in the RDSS during this time period. Mortality rates were estimated for infants (<1 year) and children younger than age 5 by Kaplan–Meier (K–M) survival estimates of incidence (mortality) rates and were expressed per 1,000 PYO. Trend tests (Chi-squared) were used to determine the significance of any mortality gradient across wealth quintiles. Concentration index and PPR were estimated as measures of health inequality.

Univariate and multivariate Poisson regression analysis were used to determine the association between SES, maternal characteristics, and mortality in children younger than age 5 and infants. Potential confounders such as mothers’ education, age, and occupation were controlled for in the multivariate model. This was based on their significance in the univariate analysis. Corresponding *P* values were calculated to test for statistical significance at the 5% level. We used STATA 10.0 by StatCorp for our statistical analysis.

Ethical approval was obtained from the University of the Witwatersrand's Committee for Research on Human Subjects and the Ifakara Health Research and Development Centre Institutional Review Board.

## Results

Socio-demographic characteristics of children, mothers of children, and household heads are presented in Table 1. In 2005, data were available for 11,189 children younger than age five living in 7,298 households contributing 9,342 person-years. There was a similar proportion of boys (49.9%) and girls (50.1%) during the period under study. Approximately one out of three households was headed by a female. Slightly less than half (46.7%) of the heads of household had primary education compared to a third (33.6%) without any education. The ages of mothers ranged from 14 to 47, with a mean age of 26.6 years (SD 7.8). The majority of mothers were 21–29 years old (*n*=4,454; 39.8%). A total of 5,695 (51%) had attained primary education, whilst 4,777 (43%) had no school education.

**Table 1:** Socio-demographic characteristics of 11,189 children younger than age 5 at Rufiji Demographic Surveillance Site, 2005

Variable	Frequency	Percentage
Sex of child		
Male	5,604	50.1
Female	5,585	49.9
Age of child (years)		
< 1	2,427	21.7
1–2	2,155	19.3
2–3	2,260	20.2
3–4	2,184	19.5
4–5	2,163	19.3
Under-five SES		
Poorest	2,251	20.1
Poorer	2,246	20.1
Poor	2,218	19.8
Less poor	2,239	20.0
Least poor	2,235	20.0
Household head sex		
Male	7,750	69.3
Female	3,439	30.7
Household head education		
No education	3,726	33.6
Primary education	5,221	46.7
Secondary education	470	4.2
Non-formal education	537	4.8
Others	1,199	10.7
Household head marital status		
Not married	972	8.7
Married	7,837	63.1
Widow/divorced/separated	1,142	10.1
Other	1,238	11.1
Household head occupation		
Not employed	151	1.4
Farming/animal husbandry	7,096	63.3
Casual worker	2,563	22.9
Student	28	0.3
Others	1,351	12.1
Maternal age (years)		
Under 20	2,791	24.9
21–29	4,454	39.8
30 +	3,648	32.6
Missing	296	2.7
Maternal education		
No education	4,777	42.7
Primary education	5,695	50.9
Secondary education	376	3.4
Non-formal education	43	0.4
Missing	298	2.6
Maternal marital status		
Not married	1,905	17.0
Married	7,180	69.8
Widow/divorced/separated	1,089	9.7
Other	89	0.8
Missing	296	2.7
Maternal occupation		
Not employed	763	6.8
Farming/animal husbandry	8,457	75.6
Casual worker	1,710	15.3
Student	87	0.8
Others	172	1.5

The relationship between SES and overall under-five mortality is summarised in Table 2. Mortality rates were shown to be highest in the poorest quintile [40.7 per 1,000 PYO; 95% confidence interval (CI) (32.6–50.9)] and lowest in the least poor quintile [17.1 per 1,000 PYO; 95% CI (12.1–24.2)]. In general, mortality rates decreased as wealth index quintile increased. Children in the poorest quintile were 140% more likely to die before reaching their fifth birthday than those in the least poor households. There was a statistically significant inverse trend (*P*<0.001). The mortality concentration index of −0.16 [95% CI (−0.24, −0.08)] showed a pro-poor concentration of under-five mortality.

**Table 2:** Under-five (<5 years) mortality rates by wealth quintile at Rufiji Demographic Surveillance Site, 2005 with inequality measures

Quintile	*N*	Under-five person years observed (PYOs)	Deaths (<5 years)	Under-five mortality rate/1,000 PYOs (95% CI)
1st (poorest)	2,251	1,891.6	77	40.7 (32.6–50.9)
2nd (poorer)	2,246	1,878.1	53	28.2 (21.6–36.9)
3rd (poor)	2,218	1,846.7	48	25.9 (19.6–34.5)
4th (less poor)	2,239	1,857.4	41	22.1 (16.3–30.0)
5th (least poor)	2,235	1,867.7	32	17.1 (12.1–24.2)
Total	11,189	9,341.6	251	26.9 (23.7–30.4)
		Chi-square trend		*P*<0.001
		Poorest–least poor ratio		2.4
		Concentration index		−0.16 (−0.24, −0.08)

Table 3 shows results from the relationship between SES and mortality from Poisson regression for children younger than age 5 with relative risks described as incident rate ratio. In univariate Poisson regression, children in the least poor households were shown to have a 58% significantly reduced risk of dying as compared to the poorest households [crude incidence rate ratio (IRR) = 0.42; *P*<0.001; 95% CI (0.27–0.62)]. Subsequent adjustment for maternal education, maternal age, and maternal occupation only marginally attenuated the observed association between SES and under-five mortality [adjusted IRR = 0.48; *P*=0.002; 95% CI (0.30–0.80)]. Chi-squared test for trend across wealth index quintiles was significant for children younger than age 5 (*P*<0.001). Children younger than age 5 whose mothers had attained secondary education had a 70% reduced mortality risk [adjusted IRR = 0.30; *P*=0.006; 95% CI (0.22–0.88)] and those whose mothers had attained primary education had a 24% reduced mortality risk [adjusted IRR = 0.76; *P*=0.008; 95% CI (0.62–0.90)] compared to those whose mothers had not attained any formal education after adjusting for SES, maternal age, and maternal occupation.

**Table 3:** Univariate and multivariate analysis of risk factors for under-five (<5 years) mortality at Rufiji Demographic Surveillance Site, 2005

Variable	(Unadjusted) IRR	95% CI	*P*	(Adjusted) IRR*	95% CI	*P*
Wealth index						
Poorest (reference)	1			1		
Poorer	0.69	0.48–0.98	0.04	0.82	0.55–1.30	0.37
Poor	0.63	0.45–0.92	0.02	0.66	0.43–1.03	0.68
Less poor	0.54	0.37–0.79	0.00	0.61	0.33–0.39	0.03
Least poor	0.42	0.27–0.63	0.00	0.48	0.30–0.80	0.00
Sex of child						
Male (reference)	1					
Female	0.81	0.63–1.03	0.08			
Maternal education						
No education (reference)	1			1		
Primary	0.70	0.52–0.95	0.02	0.76	0.62–0.90	0.01
Secondary	0.23	0.06–0.93	0.03	0.30	0.22–0.88	0.01
Mothers age (years)						
Under 20 (reference)		1		1		
21–29	0.86	0.63–1.19	0.37	0.84	0.58–1.23	0.39
30 +	1.03	0.75–1.42	0.86	0.94	0.64–1.40	0.80
Marital status						
Married (reference)	1					
Not married	1.38	0.97–1.98	0.07			
Divorced/separated	1.80	0.82–2.06	0.08			
Other	1.86	0.51–5.35	0.39			
Maternal occupation						
Not employed (reference)	1			1		
Farming	0.58	0.34–0.98	0.04	0.56	0.30–1.04	0.07
Casual worker	0.53	0.28–0.98	0.04	0.62	0.32–1.20	0.16
Student	1.23	0.36–4.25	0.74	1.31	0.36–4.80	0.68
Other	0.96	0.35–2.65	0.94	0.83	0.29–2.30	0.73

IRR = incidence rate ratio.

* Adjusted for maternal education, maternal age and occupation.

Subsequent analysis of infant mortality rates across the different wealth quintiles showed similar results to under-five mortality in Table 4, with infant mortality rate to be highest in the poorest quintile at 158.5 per 1,000 PYO [95% CI (114.9–218.8)] and lowest in the least poor quintile at 106.3 per 1,000 PYO [95% CI (69.3–163.1)]. Children in the poorest households were about 50% more likely to die during infancy than those in the least poor as reflected in the poorest to least poor ratio of 1.5. A mortality concentration index of −0.07 [95% CI (−0.13, −0.0003)] for infants is an indication of excess mortality in poorest infants household, confirming the difference in infant mortality rates amongst the poorest and the least poor.

**Table 4:** Infant (0–1 year) mortality rate by wealth quintile at Rufiji Demographic Surveillance Site, 2005 with inequality measures

Quintile	*N*	Infants person years observed (PYOs)	Deaths (0–1 year)	Infant mortality rate/1,000 PYOs (95% CI)
1st (poorest)	495	233.4	37	158.5 (114.9–218.8)
2nd (poorer)	521	234.4	26	110.9 (75.5–162.9)
3rd (poor)	491	221.4	27	122.0 (83.6–177.8)
4th (less poor)	469	207.1	24	115.9 (77.7–172.9)
5th (least poor)	451	197.5	21	106.3 (69.3–163.1)
Total	2,427	1,093.8	135	123.4 (104.3–146.1)
		Chi-square trend		*P*=0.1
		Poorest–least poor ratio		1.5
		Concentration index		−0.07 (−0.13, −0.0003)

## Discussion

The current study investigated the relationship between household SES, health equity, and under-five mortality in a rural area in Tanzania. Presence of significant socio-economic inequality in under-five mortality at RDSS was observed, adding to the body of evidence regarding the important role of SES in under-five mortality. Measures employed were PCA to derive a wealth index as a proxy for SES, and the use of the PPR and the mortality concentration index to reflect health inequality. PCA has previously been shown to provide a reliable index reflecting combined information on household assets (20). Internal consistency of the constructed wealth index was assessed by examining its distribution against the wealth quintiles of the household variables that had been used for its creation. Patterns on how the asset and household variables change with the quintiles were as expected indicating sensitivity of the PCA approach to differences in SES. The gradients are sufficient to predict health outcomes such as under-five mortality even in a source population that appears to be broadly homogenous with regard to poverty. The derived PPR value of 1.5 for children younger than age 5, contrasting children in the first (least poorest) to the fifth (poorest) wealth quintile, and a mortality concentration index of −0.16 in the least poor households indicate disproportionate concentration of under-five mortality among the poor and hence health inequity in the current study.

The method employed in the current study to compute mortality rates used person-years as a reference and differs from the method used in DHS reports in Tanzania using live births as the reference category. Subsequently, we report mortality rates for infants and children younger than age 5 that differ from previous DHS reports from Tanzania. The Pwani region, where the RDSS is located, is characterised by a high malaria prevalence [21% in children younger than age 5 as reported in the 2007–08 Tanzania HIV and Malaria Indicator Survey (21)] and a HIV prevalence of 7.3% in adults and 0.9% in children younger than 15 years of age (22). High malaria incidence and the ongoing HIV epidemic in combination with low access to high-quality maternal health care may have contributed to the high infant mortality discussed in this study.

### Factors associated with under-five mortality

In previous research, it has been suggested that children born to poor mothers in rural areas face great challenges of survival (23). They are often born at home, without any contact with the health system. An analysis of 50 developing countries found that children born to mothers in the poorest fifth of a population were almost 30% more likely to die as compared to those in the richest fifth (24). Children born to mothers in rural areas in Burkina Faso were more likely to die compared to those in the urban areas (25). Disparities within some countries are especially dramatic. For example, in India, children born to the poorest mothers died at a rate that is 56% higher than babies born to the richest mothers, and in Bolivia, the newborn mortality rate was 70% higher among the poor (24).

Previous studies conducted in South Africa (26–28), Kenya (29), and Nigeria (30) observed higher SES to protect against child mortality and are thus very consistent with the findings of the current study, providing further evidence that wealth inequality is an important risk factor of child mortality. Appropriately identifying disparities in health outcomes is essential, as it informs policy makers about groups that are in greater need of assistance. Even though the population we studied could have been easily identified as homogenously poor, we have shown that it is not. This has important implications for policies aimed at improving child health outcomes amongst the poor as even in this context trade-offs between universal coverage and targeting health interventions may have to be considered.

Children of mothers with primary (and secondary) education have a significant decreased mortality risk compared to those of mothers with no education. Likewise, mothers who missed out on schooling are more likely to be poor, to get pregnant younger, and more often, to have more children, to be less knowledgeable about family planning and HIV prevention and to be unprepared to look after the health and wellbeing of their babies (31). Mothers with less education are furthermore less likely to receive skilled medical care during pregnancy and childbirth. In Egypt, for example, only 33% of women with no education receive any prenatal care, and only 17% receive regular prenatal care, while 75% of women with secondary or higher education receive prenatal care and 60% receive regular care (32). Educated women are more likely to be mothers who are healthy, well nourished, economically empowered and resourceful when it comes to caring for themselves and their babies. Women being educated may in fact be a product of a broader social transformation and not just a predictor of child well-being independent of SES. There is the need to further explore the social determinants of women's education and child health.

The potential limitation of this analysis is that it does not adjust for the weight of the child at birth due to RDSS having no data on birth weight. In previous research (32), it has been suggested that children with low birth weight are at increased risk of dying. At the same time, low birth weight has been shown to be associated with low SES. Likewise, family size and distance to health facility were observed to be related to both SES and under-five mortality; however, unfortunately these data were not also available for this analysis (11).

## Conclusion

Household socio-economic inequality and maternal education were observed to be strongly associated with under-five mortality in rural Tanzania. Our study adds to the understanding of the drivers of under-five mortality in rural settings, suggesting that targeted interventions to address these factors would likely contribute towards a reduction of child mortality in rural Tanzania.
